# Supplementary material for: Peripheral apoptosis and limited clonal deletion during physiologic murine B lymphocyte development
Source: Nat Commun. 2024 Jun 1;15:4691. doi: 10.1038/s41467-024-49062-x (PMC11144239; doi:10.1038/s41467-024-49062-x)
Supplement: Supplementary file 1 — Supplementary Information [file 41467_2024_49062_MOESM1_ESM.pdf]

Supplementary Information

Peripheral apoptosis and limited clonal deletion during physiologic murine B lymphocyte development

Mikala JoAnn Simpson[1,#], Anna Minh Newen[1,#], Christopher McNees[1], Sukriti Sharma[1], Dylan Pfannenstiel[1], Thomas Moyer[2], David Stephany[2], Iyadh Douagi[2], Qiao Wang[3], and Christian Thomas Mayer[1,*]

[1]Experimental Immunology Branch, Center for Cancer Research, National Cancer Institute, National Institutes of Health, Bethesda, MD, USA

[2]Flow Cytometry Section, Research Technologies Branch, National Institute of Allergy and Infectious Diseases, National Institutes of Health, Bethesda, MD, USA

[3]Key Laboratory of Medical Molecular Virology (MOE/NHC/CAMS), Shanghai Institute of Infectious Disease and Biosecurity, School of Basic Medical Sciences, Fudan University, Shanghai, China

[#]Equally contributing authors

*Corresponding author; Email: christian.mayer@nih.gov

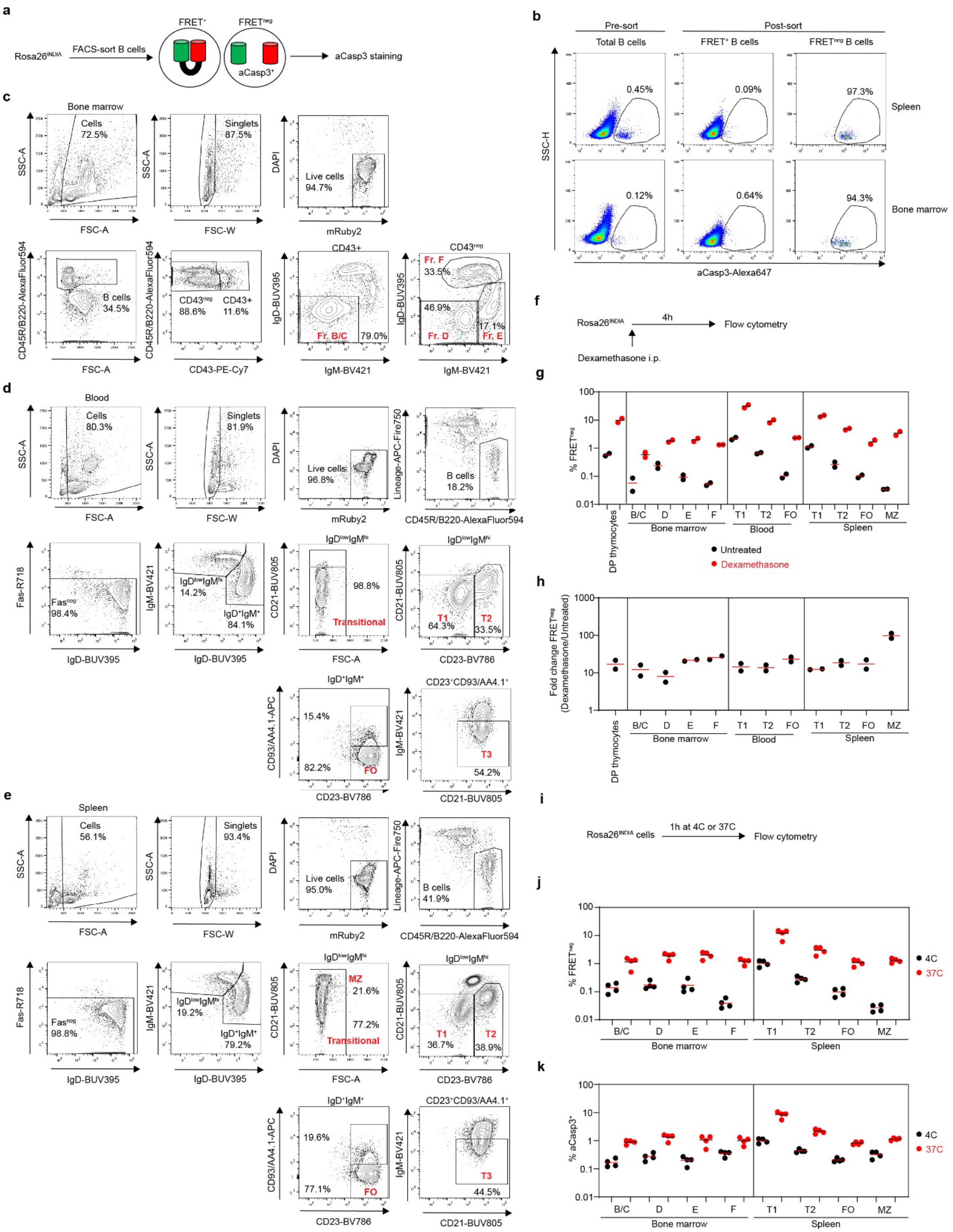

Supplementary Fig. 1. Quantitation of apoptosis during physiologic B cell development.

a, b $FRET^+$ and $FRET^{neg}$ B cells were FACS sorted from the bone marrow and spleen of $Rosa26^{INDIA}$ mice. Gating was done as in Supplementary Fig. 1e until the fifth contour plot except that GL7 was used instead of CD95 to exclude germinal center B cells from the spleen. Immediately after FACS sorting, dead/necrotic cells were stained with Zombie NIR, followed by intracellular staining of early apoptotic cells with active caspase-3 (aCasp3) and analysis by flow cytometry. Pre-sort B cells served as control. **a** Schematics of the FACS-sorting experiment. **b** aCasp3 staining and side scatter (SSC-H) is shown for indicated Zombie NIR^{neg} B cell populations before and after sorting. Percentages of $aCasp3^+$ cells are shown. One of two independent experiments with similar results is shown. **c-e** $Rosa26^{INDIA}$ mice were analyzed by flow cytometry. Gating strategy is shown for **c** bone marrow, **d** blood, and **e** spleen (Fr. B/C: pro/pre-B cells, Fr. D: small pre-B cells, Fr. E: immature B cells, Fr. F: mature recirculating B cells, T1: transitional 1 B cells, T2: transitional 2 B cells, T3: anergic B cells, FO: mature follicular B cells, MZ: marginal zone B cells). Representative plots are shown from one out of three experiments with similar results each including one or two animals. **f-h** $Rosa26^{INDIA}$ mice were injected intraperitoneally with 0.5mg dexamethasone and analyzed by flow cytometry 4h later. Untreated mice served as controls. **f** Experimental scheme. **g** Quantitation of $FRET^{neg}$ cells in untreated (black circles) or dexamethasone treated (red circles) mice among the indicated subsets and tissues. B cell subsets were gated as described in **c-e**. As additional control, double positive thymocytes (DP) were analyzed and were gated $DAPI^{neg}$Lineage(F4/80, CD19, NK1.1, Ly-6G, Ter-$119)^{neg}$$mRuby2^+CD4^+CD8\alpha^+TCR\beta^{low}$. **h** Comparison of the fold changes in the fraction of $FRET^{neg}$ cells upon dexamethasone treatment. **f-h** Two experiments were combined with one animal per condition. **i-k** $Rosa26^{INDIA}$ bone marrow and spleen cells were cultured for 1h at 37°C (red circles) or were kept for 1h at 4°C as control (black circles), followed by flow cytometry. **i** Experimental scheme. **j, k** Samples were fixed and intracellularly stained for aCasp3 to allow direct comparison of **j** $FRET^{neg}$ and **k** $aCasp3^+$ cells. Two experiments were combined with one to three animals per condition. B cell subsets were gated as described in **c-e** except that CD93/AA4.1 was not used and T3 cells were not discriminated from mature FO B cells. Source data are provided as a Source Data file.

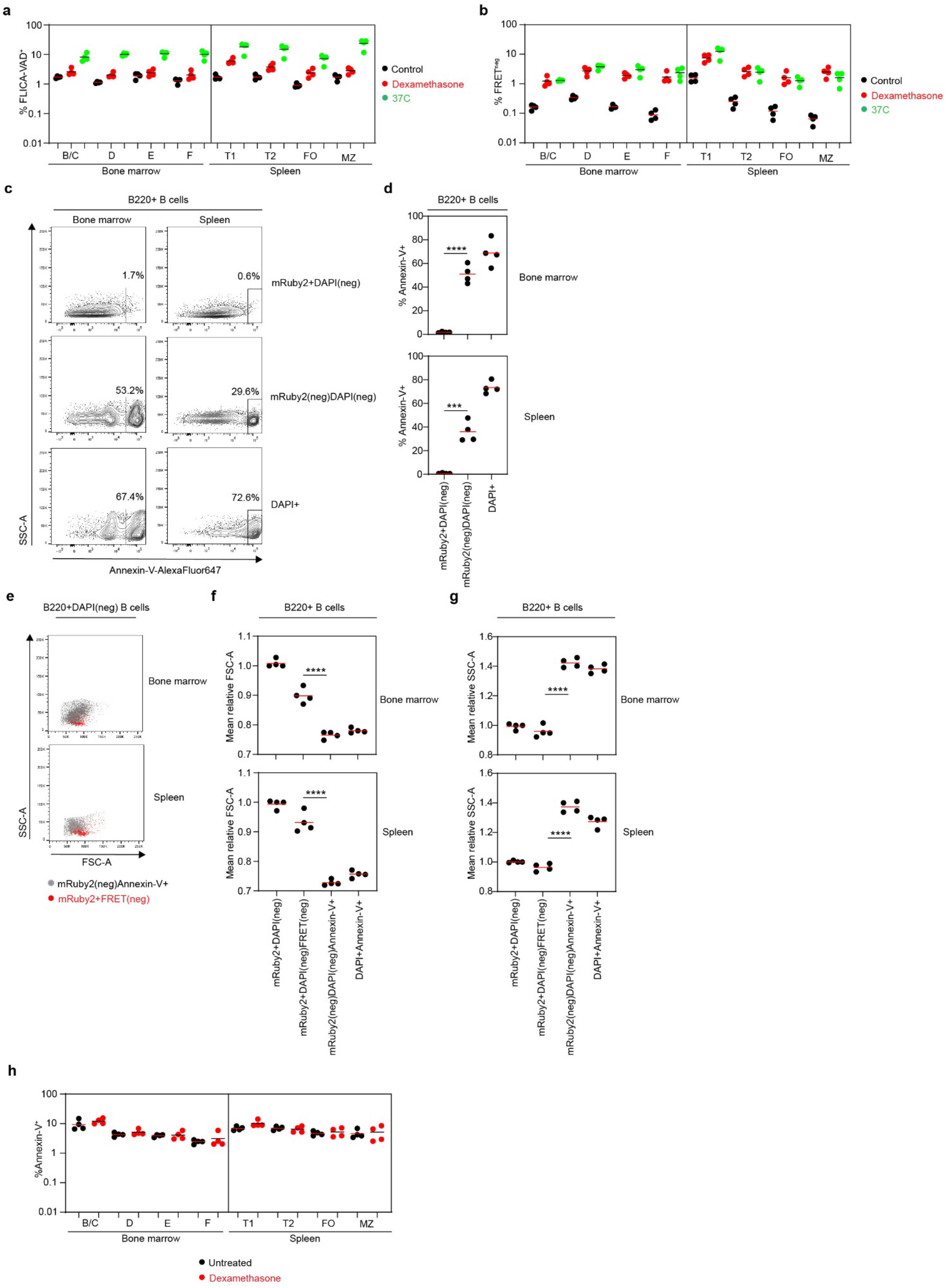

a
% FLICA-VAD+
Control
Dexamethasone
37C
B/C
D
E
F
T1
T2
FO
MZ
Bone marrow
Spleen
b
% FRETneg
c
B220+ B cells
Bone marrow
Spleen
1.7%
0.6%
mRuby2+DAPI(neg)
53.2%
29.6%
mRuby2(neg)DAPI(neg)
67.4%
72.6%
DAPI+
SSC-A
Annexin-V-AlexaFluor647
d
% Annexin-V+
e
B220+DAPI(neg) B cells
FSC-A
mRuby2(neg)Annexin-V+
mRuby2+FRET(neg)
f
Mean relative FSC-A
mRuby2+DAPI(neg)FRET(neg)
mRuby2(neg)DAPI(neg)Annexin-V+
DAPI+Annexin-V+
g
Mean relative SSC-A
h
%Annexin-V+
Untreated

Supplementary Fig. 2. Analysis of FLICA-VAD and Annexin-V staining during B cell development.

Rosa26^{INDIA} mice were injected with 0.5mg dexamethasone intraperitoneally 4 hours prior to analysis by flow cytometry. Untreated Rosa26^{INDIA} mice served as controls. For some experiments, untreated cells were cultured for 1h at 37°C whereas control cells or dexamethasone treated cells were kept at 4°C during that time. Comparison of **a** FLICA-VAD^{+} or **b** FRET^{neg} cells among the indicated B cell subsets and conditions. **c-h** Annexin-V staining. **c** Representative contour plots show Annexin-V and SSC-A profiles of indicated B cell subsets. **d** Quantitation of Annexin-V^{+} cells among the indicated B cells in bone marrow and spleen. **e** Representative dot plots show forward scatter (FSC-A) and side scatter (SSC-A) profiles of the indicated B cell subsets. **f, g** Quantitation of **f** mean relative FSC-A or **g** mean relative SSC-A among the indicated B cell subsets. Values were normalized to mRuby2^{+}DAPI^{neg} cells. **h** Comparison of Annexin-V^{+} cells among the indicated B cell subsets and conditions. B cell subsets were gated as described in Supplementary Fig. 1c, e except for not gating mRuby2^{+} cells which excludes Annexin-V^{+} cells. **a-h** Results are combined from two independent experiments (n=4 mice) with similar results (**** $p<0.0001$, *** $p=0.0002$, unpaired two-tailed t-test). Source data are provided as a Source Data file.

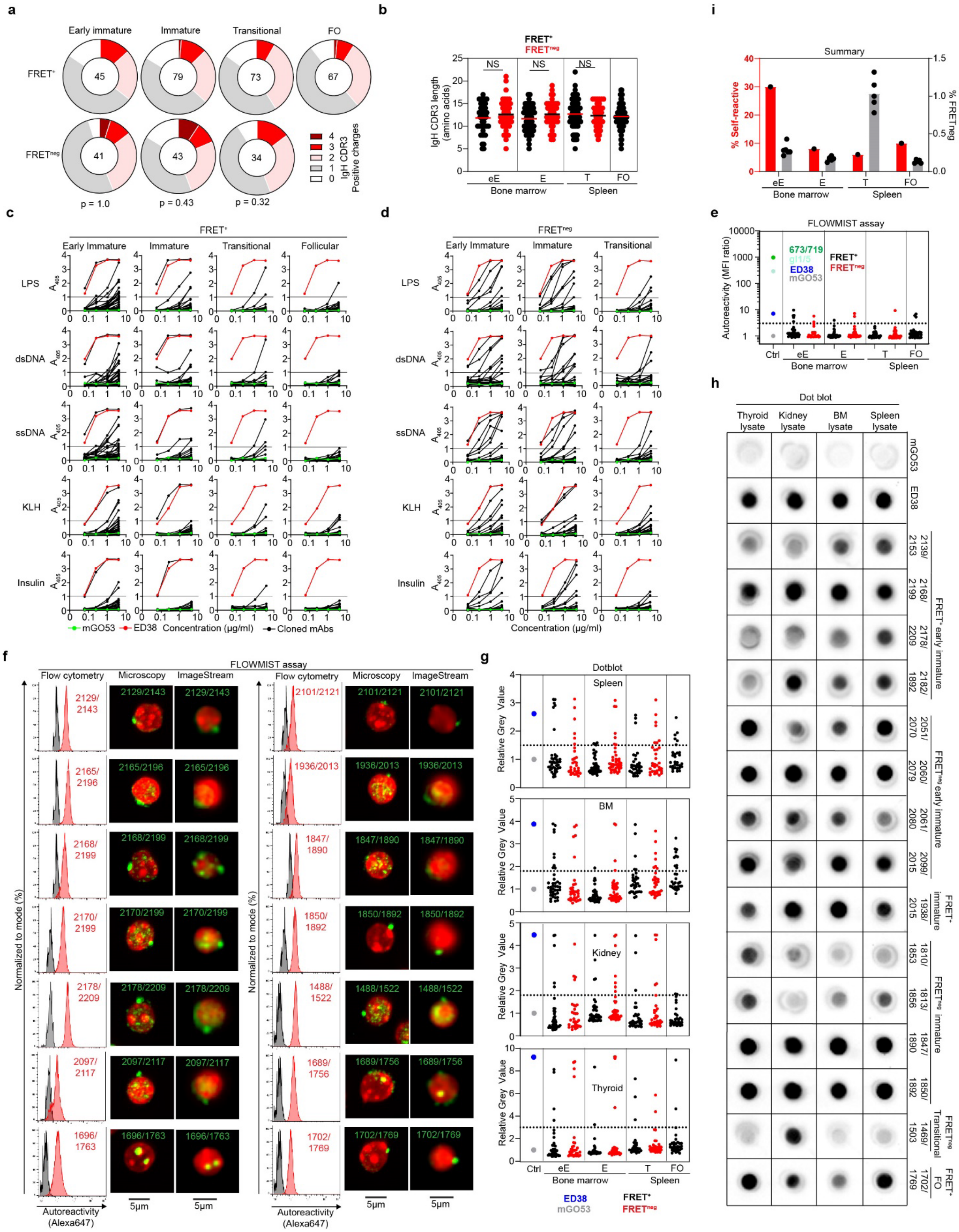

Supplementary Fig. 3. Limited clonal deletion of autoreactive and polyreactive B cells during physiologic B cell development.

Live ($FRET^+$) and apoptotic ($FRET^{neg}$) B cells of various developmental stages were single-cell FACS-sorted from the bone marrow (eE: early immature, E: immature) and spleen (T: transitional, FO: mature follicular) of $Rosa26^{INDIA}$ mice. Ig genes were PCR amplified, sequenced, cloned into expression vectors and the corresponding recombinant antibodies were expressed, purified, and tested. **a, b** Ig sequence analysis of indicated B cell compartments. Only cells with paired functional heavy- and light chains were considered for analysis. **a** Number of positively charged amino acids in the IgH complementarity-determining region 3 (CDR3). Numbers in the center of each pie indicate the numbers of B cell receptors (BCR) assessed. The p value for comparing IgH CDR3s with at least three positive charges among $FRET^+$ and $FRET^{neg}$ cells are shown below the pie charts (two-tailed Fisher's exact test). **b** IgH CDR3 lengths. Each dot represents one cloned BCR (n=45 BCRs for $FRET^+$ eE, n=41 BCRs for $FRET^{neg}$ eE, n=79 BCRs for $FRET^+$ E, n=43 BCRs for $FRET^{neg}$ E, n=73 BCRs for $FRET^+$ T, n=34 BCRs for $FRET^{neg}$ T, n=67 BCRs for $FRET^+$ FO). Horizontal bars: mean values (ns, not statistically significant; unpaired two-tailed Mann-Whitney test). **c, d** Enzyme-linked immunosorbent assay (ELISA) measurements show binding of monoclonal antibodies (mAb) cloned from the indicated **c** $FRET^+$ and **d** $FRET^{neg}$ B cell compartments (black lines) to LPS, double-stranded DNA (dsDNA), single-stranded DNA (ssDNA), keyhole limpet hemocyanin (KLH) and insulin. mGO53 was included as nonreactive control antibody (green lines), ED38 was included as highly polyreactive control antibody (red lines). **e, f** Autoreactivity determined by the flow cytometry-based screening assay coupled with confocal microscopy or imaging flow cytometry (FLOWMIST). **e** Summary of mAb screening for autoreactivity by flow cytometry. Fold mean fluorescence intensity (MFI) relative to the negative control mGO53 is plotted. Dotted line represents the cutoff for positive reactivity. Control antibodies were included for comparison (mGO53: nonreactive, ED38: polyreactive, 673/719 and its germline revertant gl1/5: anti-nuclear). Reactive antibodies were confirmed by at least two independent experiments with similar results. **f** All autoreactive mAbs identified in **e** are shown. Flow cytometry histograms for each mAb (red) are overlaid with the nonreactive mGO53 control antibody (gray). Confocal microscopy and ImageStream analysis depict the autoreactive fluorescence pattern (green) and DAPI (red). 5µm scale bars are shown on the bottom. **g, h** Antibody screening for autoreactivity against C57Bl/6J spleen, bone marrow, kidney, and thyroid lysates by dot blot. mGO53 (nonreactive) and ED38 (highly polyreactive) antibodies were included as controls. Samples derive from parallel blots of the same experiment. **g** The relative gray value for the dot area of each antibody is shown normalized to nonreactive mGO53 antibody (gray). Horizontal lines indicate cutoff for positive reactivity. **h** Dot blots for mAbs reacting to at least one tissue are shown. Each reactive antibody was confirmed in two independent experiments with similar results. **i** The summary graph plots the measured percentage of self-reactive $FRET^+$ B cells (red bars represent mean; from Fig. 4g) and the measured percentages of $FRET^{neg}$ B cells (grey bars represent mean; from Fig. 1e) across B cell development. **a-i** Monoclonal antibodies are derived from two to three independent single-cell sorts for each compartment. **f, h** Images were cropped to depict centered individual cells from micrographs or individual wells from 96-well dot blot images and were grouped by antibody. Source data are provided as a Source Data file.

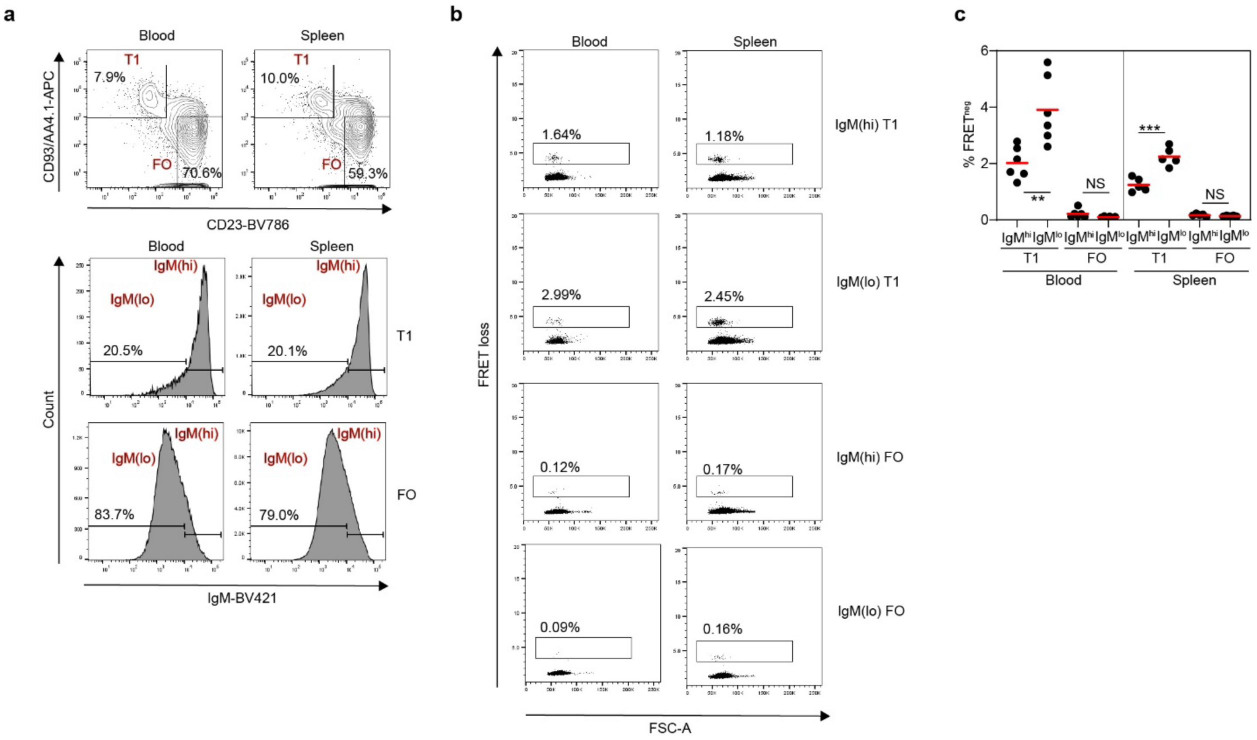

Supplementary Fig. 4. Increased apoptosis of peripheral IgM^{lo} T1 B cells.

Rosa26^{INDIA} mice were analyzed by flow cytometry. Blood and spleen B cells were gated CD45R/B220^{+}mRuby2^{+}DAPI^{neg}Lineage(CD4, CD8α, F4/80, NK1.1, Ly-6G, Ter-119)^{neg}CD95^{neg} as shown in Supplementary Fig. 1d, e. **a** Gating of CD93/AA4.1^{+}CD23^{neg} transitional 1 (T1) and CD93/AA4.1^{neg}CD23^{+} mature follicular B cells (FO). IgM^{lo} and IgM^{hi} T1 and FO B cell subsets were distinguished. **b** Representative dot plots show FRET loss in the indicated B cell subsets. **c** Quantitation of FRET loss in the indicated B cell subsets. Data are combined from three independent experiments with similar results (Blood: n=6 mice; Spleen: n=5 mice) (*** p = 0.0006, ** p = 0.0059, not statistically significant (ns); unpaired two-tailed t-test). Source data are provided as a Source Data file.

a

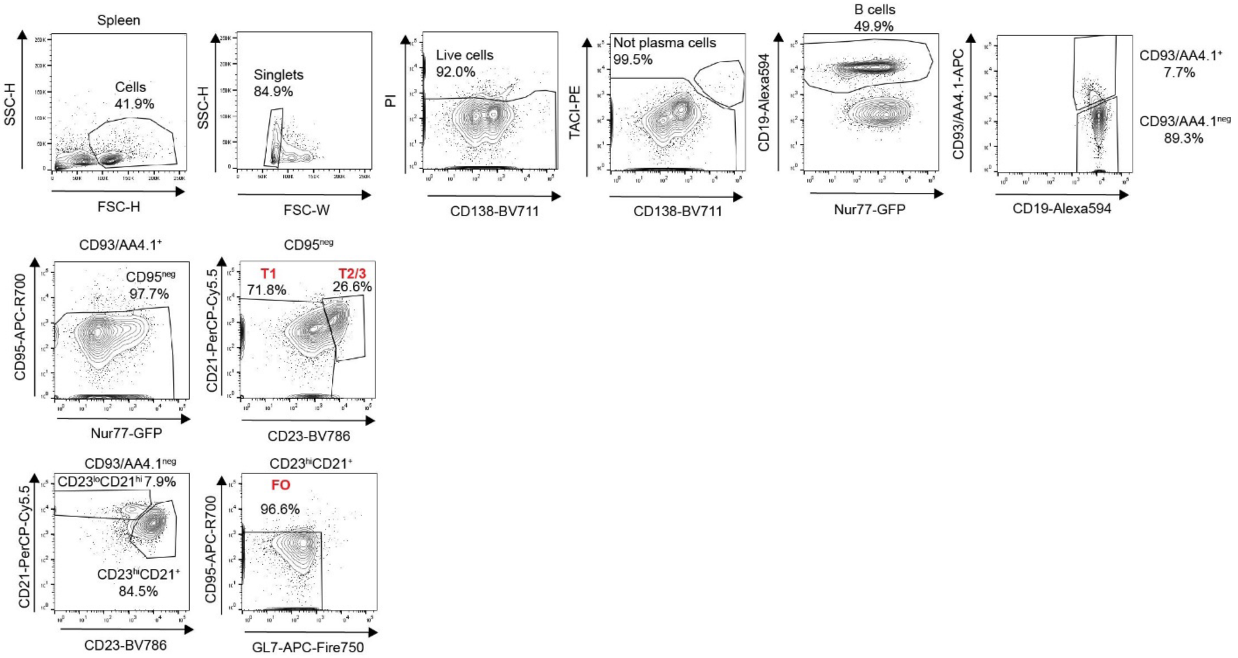

b

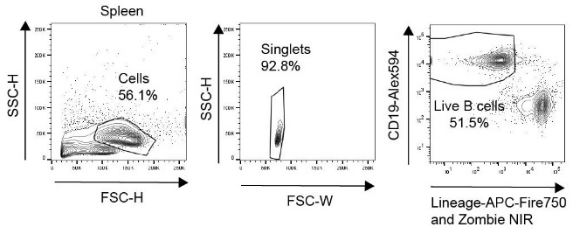

Supplementary Fig. 5. Gating strategy for Figure 5.

a Gating strategy for Figure 5a-d. **b** Gating of B cells for Figure 5e-j. Subsequent gating was done as in **a** except that GL7 was not used.

Antigen	Antibody clone	Conjugate	Supplier	Catalog #	Dilution
Active caspase-3	C92-605	AlexaFluor647	BD	560626	1:40
Bcl-2	10C4	eFluor450	Thermo Fisher	48-6992-42	1:20
CD3ε	145-2C11	PE	BD	553064	1:80
CD4	RM4-5	Biotin	BioLegend	100508	1:200
CD4	RM4-5	APC-eFluor780	Thermo Fisher	47-0042-82	1:200
CD8α	53-6.7	Biotin	BD	553029	1:200
CD8α	53-6.7	APC-eFluor780	Thermo Fisher	47-0081-82	1:200
CD16/CD32	2.4G2	Unlabeled	In house	N/A	1:10
CD19	6D5	AlexaFluor594	BioLegend	115552	1:800
CD19	6D5	BV605	BioLegend	115540	1:200
CD21/CD35	7G6	BUV805	BD	741961	1:800
CD21/CD35	7G6	PerCP-Cy5.5	BD	562797	1:200
CD21/CD35	7G6	BV510	BD	747764	1:200
CD23	B3B4	BV786	BD	563988	1:200
CD38	90	AlexaFluor700	Thermo Fisher	56-0381-82	1:200
CD43	S7	PE-Cy7	BD	562866	1:160
CD43	S7	PerCP-Cy5.5	BD	562865	1:25
CD45R/B220	RA3-6B2	AlexaFluor594	BioLegend	103254	1:400
CD45R/B220	RA3-6B2	AlexaFluor647	Thermo Fisher	RM2621	1:400
CD93/AA4.1	AA4.1	APC	Thermo Fisher	17-5892-83	1:100
CD93/AA4.1	AA4.1	PE	BioLegend	136504	1:40
CD95/Fas	Jo2	APC-R700	BD	565130	1:400
CD95/Fas	Jo2	R718	BD	752226	1:3200
CD95/Fas	Jo2	PE-Cy7	BD	557653	1:800
CD138	281-2	BV711	BioLegend	142519	1:8000
CD267/TACI	ebio8F10-3	PE	Thermo Fisher	12-5942-81	1:200
F4/80	BM8	Biotin	Thermo Fisher	13-4801-85	1:100
F4/80	BM8	APC-eFluor780	Thermo Fisher	47-4801-82	1:100
GL7	GL7	Biotin	BioLegend	144616	1:3200
GL7	GL7	eFluor660	BioLegend	144616	1:400
IgD	11-26c.2a	BUV395	BD	564274	1:100
IgM	RMM-1	BV421	BioLegend	406517	1:10
IgM	II/41	PE-Cy7	Thermo Fisher	25-5790-82	1:100
IgM	II/41	eFluor660	Thermo Fisher	50-5790-82	1:25
Ly-6G	1A8	Biotin	BioLegend	127604	1:100
NK1.1	PK136	Biotin	BD	553163	1:200
NK1.1	PK136	APC-eFluor780	Thermo Fisher	47-5941-82	1:200
TER-119	TER-119	Biotin	Thermo Fisher	13-5921-82	1:400

Supplementary Table 1. Details for monoclonal antibodies used in this study.

Supplementary Fig. 6

Uncropped blots related to Supplementary Fig. 3h

M71 Spleen lysate Blot 1

Original Blot

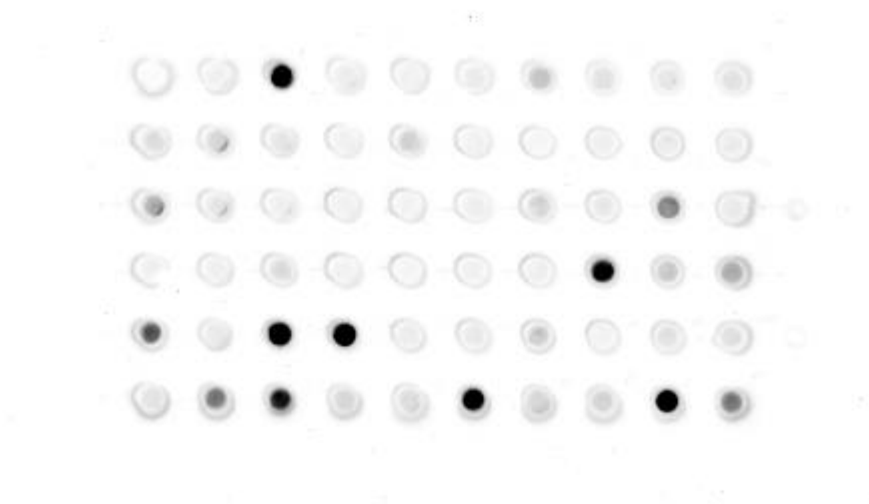

Primary antibody:
Indicated monoclonal antibodies
(human IgG1 constant region)

Secondary antibody:
Peroxidase-conjugated goat anti-human IgG Fc

Layout of primary antibodies

empty	mGO53	ED38	1464<br>1498	1467<br>1501	1468<br>1502	1469<br>1503	1470<br>1504	1473<br>1507	1477<br>1511
1480<br>1514	1482<br>1516	1483<br>1517	1486<br>1520	1488<br>1522	1533<br>1605	1534<br>1606	1535<br>1607	1539<br>1611	1540<br>1612
1541<br>1613	1550<br>1622	1553<br>1625	1554<br>1752	1677<br>1744	1685<br>1752	1686<br>1753	1688<br>1755	1689<br>1756	1691<br>1758
1692<br>1759	1693<br>1760	1696<br>1763	1697<br>1764	1698<br>1765	1699<br>1766	1700<br>1767	1702<br>1769	1741<br>1808	1810<br>1853
1813<br>1856	1846<br>1889	1847<br>1890	1850<br>1892	1900<br>1977	1905<br>1982	1909<br>1986	1911<br>1988	1912<br>1989	1913<br>1990
1935<br>2012	1936<br>2013	1938<br>2015	1939<br>2016	1944<br>2021	2051<br>2070	2054<br>2073	2055<br>2074	2060<br>2079	2061<br>2080

Cropped wells used in Figure

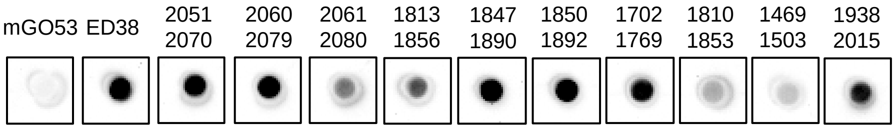

M71 Spleen lysate Blot 2

Original Blot

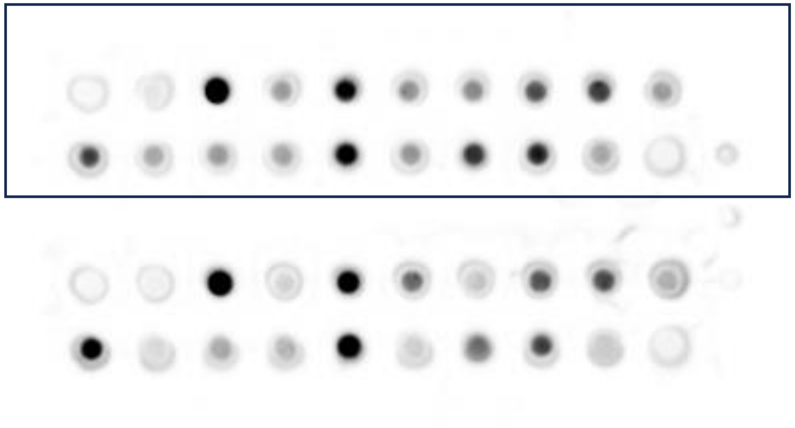

Primary antibody:
Indicated monoclonal antibodies
(human IgG1 constant region)

Secondary antibody:
Peroxidase-conjugated goat anti-human IgG Fc

Layout of primary antibodies

Empty	mGO53	ED38	2097<br>2117	2099<br>2015	2128<br>2142	2130<br>2144	2138<br>2152	2139<br>2153	2140<br>2212
2156<br>1890	2158<br>2189	2163<br>2194	2164<br>2195	2168<br>2199	2170<br>2199	2178<br>2209	2182<br>1892	2183<br>2123	

Cropped wells used in Figure

2139<br>2153	2168<br>2199	2178<br>2209	2182<br>1892	2099<br>2015

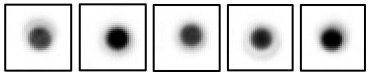

M71 Bone marrow lysate Blot 1

Original Blot

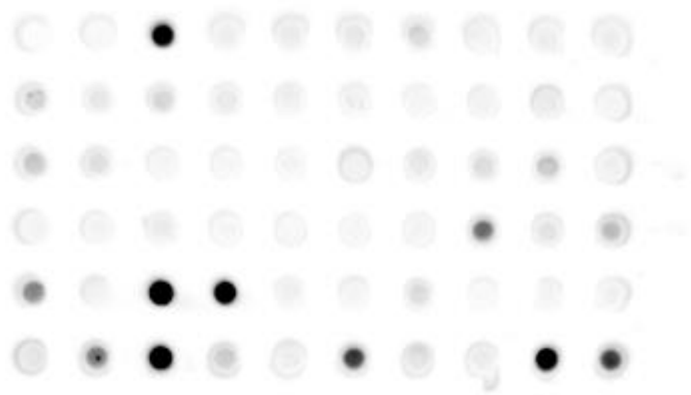

Primary antibody:
Indicated monoclonal antibodies
(human IgG1 constant region)

Secondary antibody:
Peroxidase-conjugated goat anti-human IgG Fc

Layout of primary antibodies

empty	mGO53	ED38	1464<br>1498	1467<br>1501	1468<br>1502	1469<br>1503	1470<br>1504	1473<br>1507	1477<br>1511
1480<br>1514	1482<br>1516	1483<br>1517	1486<br>1520	1488<br>1522	1533<br>1605	1534<br>1606	1535<br>1607	1539<br>1611	1540<br>1612
1541<br>1613	1550<br>1622	1553<br>1625	1554<br>1752	1677<br>1744	1685<br>1752	1686<br>1753	1688<br>1755	1689<br>1756	1691<br>1758
1692<br>1759	1693<br>1760	1696<br>1763	1697<br>1764	1698<br>1765	1699<br>1766	1700<br>1767	1702<br>1769	1741<br>1808	1810<br>1853
1813<br>1856	1846<br>1889	1847<br>1890	1850<br>1892	1900<br>1977	1905<br>1982	1909<br>1986	1911<br>1988	1912<br>1989	1913<br>1990
1935<br>2012	1936<br>2013	1938<br>2015	1939<br>2016	1944<br>2021	2051<br>2070	2054<br>2073	2055<br>2074	2060<br>2079	2061<br>2080

Cropped wells used in Figure

mGO53 ED38 2051 2070 2060 2079 2061 2080 1813 1856 1847 1890 1850 1892 1702 1769 1810 1853 1469 1503 1938 2015

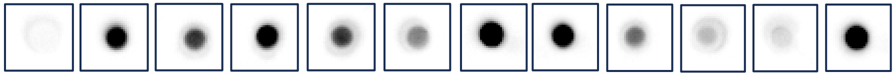

M71 Bone marrow lysate Blot 2

Original Blot

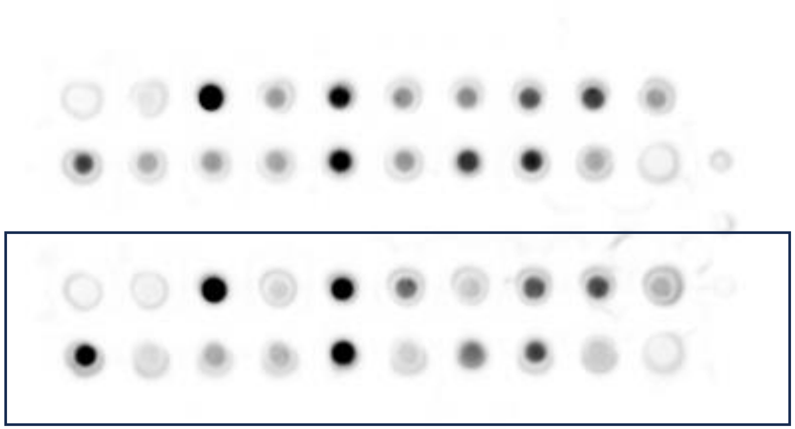

Primary antibody:
Indicated monoclonal antibodies
(human IgG1 constant region)

Secondary antibody:
Peroxidase-conjugated goat anti-human IgG Fc

Layout of primary antibodies

Empty	mGO53	ED38	2097 2117	2099 2015	2128 2142	2130 2144	2138 2152	2139 2153	2140 2212
2156 1890	2158 2189	2163 2194	2164 2195	2168 2199	2170 2199	2178 2209	2182 1892	2183 2123	

Cropped wells used in Figure

2139 2153	2168 2199	2178 2209	2182 1892	2099 2015

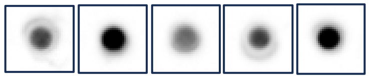

M71 Kidney lysate Blot 1

Original Blot

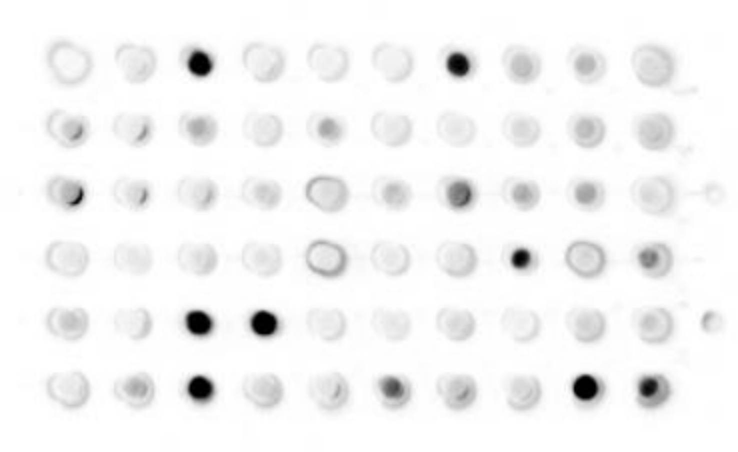

Primary antibody:
Indicated monoclonal antibodies
(human IgG1 constant region)

Secondary antibody:
Peroxidase-conjugated goat anti-human IgG Fc

Layout of primary antibodies

empty	mGO53	ED38	1464<br>1498	1467<br>1501	1468<br>1502	1469<br>1503	1470<br>1504	1473<br>1507	1477<br>1511
1480<br>1514	1482<br>1516	1483<br>1517	1486<br>1520	1488<br>1522	1533<br>1605	1534<br>1606	1535<br>1607	1539<br>1611	1540<br>1612
1541<br>1613	1550<br>1622	1553<br>1625	1554<br>1752	1677<br>1744	1685<br>1752	1686<br>1753	1688<br>1755	1689<br>1756	1691<br>1758
1692<br>1759	1693<br>1760	1696<br>1763	1697<br>1764	1698<br>1765	1699<br>1766	1700<br>1767	1702<br>1769	1741<br>1808	1810<br>1853
1813<br>1856	1846<br>1889	1847<br>1890	1850<br>1892	1900<br>1977	1905<br>1982	1909<br>1986	1911<br>1988	1912<br>1989	1913<br>1990
1935<br>2012	1936<br>2013	1938<br>2015	1939<br>2016	1944<br>2021	2051<br>2070	2054<br>2073	2055<br>2074	2060<br>2079	2061<br>2080

Cropped wells used in Figure

mGO53	ED38	2051<br>2070	2060<br>2079	2061<br>2080	1813<br>1856	1847<br>1890	1850<br>1892	1702<br>1769	1810<br>1853	1469<br>1503	1938<br>2015

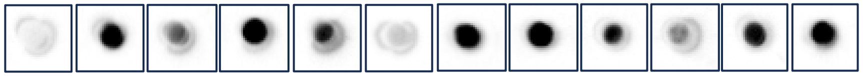

M71 Kidney lysate Blot 2

Original Blot

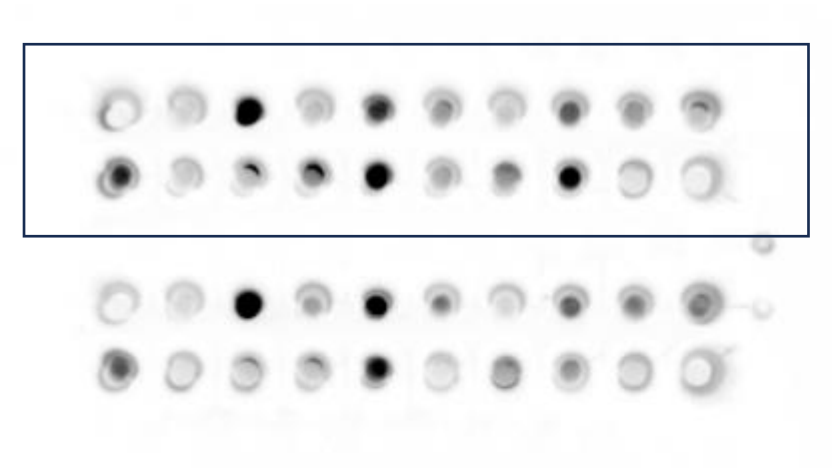

Primary antibody:
Indicated monoclonal antibodies
(human IgG1 constant region)

Secondary antibody:
Peroxidase-conjugated goat anti-human IgG Fc

Layout of primary antibodies

Empty	mGO53	ED38	2097<br>2117	2099<br>2015	2128<br>2142	2130<br>2144	2138<br>2152	2139<br>2153	2140<br>2212
2156<br>1890	2158<br>2189	2163<br>2194	2164<br>2195	2168<br>2199	2170<br>2199	2178<br>2209	2182<br>1892	2183<br>2123	

Cropped wells used in Figure

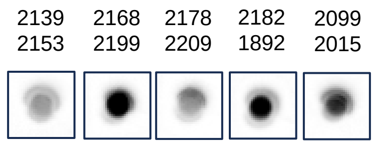

M71 Thyroid lysate 1

Original Blot

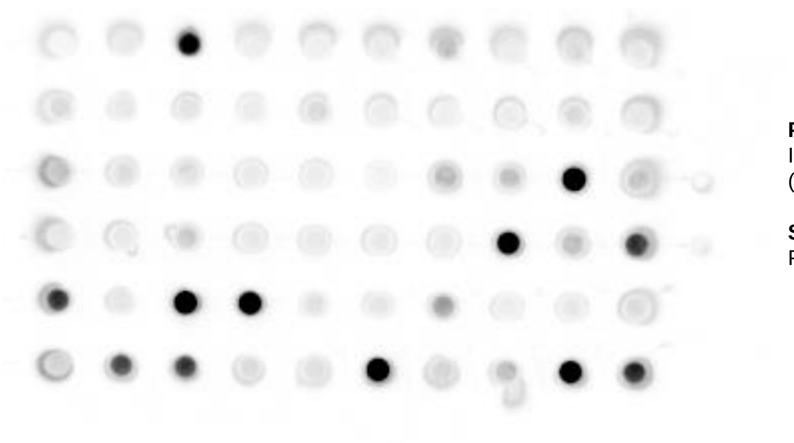

Primary antibody:
Indicated monoclonal antibodies
(human IgG1 constant region)

Secondary antibody:
Peroxidase-conjugated goat anti-human IgG Fc

Layout of primary antibodies

empty	mGO53	ED38	1464<br>1498	1467<br>1501	1468<br>1502	1469<br>1503	1470<br>1504	1473<br>1507	1477<br>1511
1480<br>1514	1482<br>1516	1483<br>1517	1486<br>1520	1488<br>1522	1533<br>1605	1534<br>1606	1535<br>1607	1539<br>1611	1540<br>1612
1541<br>1613	1550<br>1622	1553<br>1625	1554<br>1752	1677<br>1744	1685<br>1752	1686<br>1753	1688<br>1755	1689<br>1756	1691<br>1758
1692<br>1759	1693<br>1760	1696<br>1763	1697<br>1764	1698<br>1765	1699<br>1766	1700<br>1767	1702<br>1769	1741<br>1808	1810<br>1853
1813<br>1856	1846<br>1889	1847<br>1890	1850<br>1892	1900<br>1977	1905<br>1982	1909<br>1986	1911<br>1988	1912<br>1989	1913<br>1990
1935<br>2012	1936<br>2013	1938<br>2015	1939<br>2016	1944<br>2021	2051<br>2070	2054<br>2073	2055<br>2074	2060<br>2079	2061<br>2080

Cropped wells used in Figure

mGO53 ED38 2051 2070 2060 2079 2061 2080 1813 1856 1847 1890 1850 1892 1702 1769 1810 1853 1469 1503 1938 2015

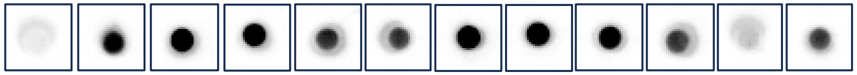

M71 Thyroid lysate 2

Original Blot

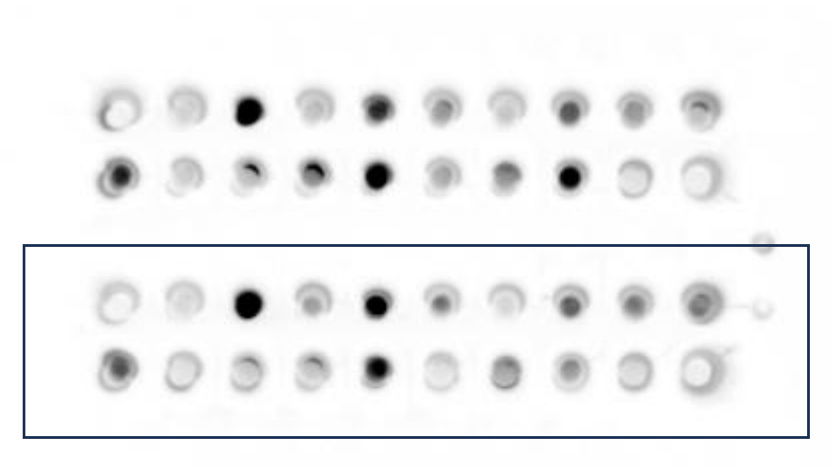

Primary antibody:
Indicated monoclonal antibodies
(human IgG1 constant region)

Secondary antibody:
Peroxidase-conjugated goat anti-human IgG Fc

Layout of primary antibodies

Empty	mGO53	ED38	2097<br>2117	2099<br>2015	2128<br>2142	2130<br>2144	2138<br>2152	2139<br>2153	2140<br>2212
2156<br>1890	2158<br>2189	2163<br>2194	2164<br>2195	2168<br>2199	2170<br>2199	2178<br>2209	2182<br>1892	2183<br>2123	

2139 2153 | 2168 2199 | 2178 2209 | 2182 1892 | 2099 2015

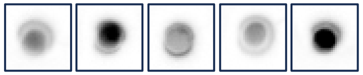

Cropped wells used in Figure
