# Supplementary material for: Peripheral apoptosis and limited clonal deletion during physiologic murine B lymphocyte development
Source: Nat Commun. 2024 Jun 1;15:4691. doi: 10.1038/s41467-024-49062-x (PMC11144239; doi:10.1038/s41467-024-49062-x)
Supplement: Supplementary file 3 — Description of Additional Supplementary Files [file 41467_2024_49062_MOESM3_ESM.pdf]

Description of Additional Supplementary Files

File Name: Supplementary Data 1
Description: Summary of cloned BCR and antibody properties

File Name: Supplementary Data 2
Description: List of primers used for BCR cloning
