# Supplementary material for: Peripheral apoptosis and limited clonal deletion during physiologic murine B lymphocyte development
Source: Nat Commun. 2024 Jun 1;15:4691. doi: 10.1038/s41467-024-49062-x (PMC11144239; doi:10.1038/s41467-024-49062-x)
Supplement: Supplementary file 6 — Reporting Summary [file 41467_2024_49062_MOESM6_ESM.pdf]

# nature portfolio

Corresponding author(s): Christian T. Mayer

Last updated by author(s): Mar 24, 2024

# Reporting Summary

Nature Portfolio wishes to improve the reproducibility of the work that we publish. This form provides structure for consistency and transparency in reporting. For further information on Nature Portfolio policies, see our Editorial Policies and the Editorial Policy Checklist.

## Statistics

For all statistical analyses, confirm that the following items are present in the figure legend, table legend, main text, or Methods section.

| n/a | Confirmed | |
|---|---|---|
| ☐ | ☒ | The exact sample size (*n*) for each experimental group/condition, given as a discrete number and unit of measurement |
| ☐ | ☒ | A statement on whether measurements were taken from distinct samples or whether the same sample was measured repeatedly |
| ☐ | ☒ | The statistical test(s) used AND whether they are one- or two-sided<br>*Only common tests should be described solely by name; describe more complex techniques in the Methods section.* |
| ☒ | ☐ | A description of all covariates tested |
| ☐ | ☒ | A description of any assumptions or corrections, such as tests of normality and adjustment for multiple comparisons |
| ☐ | ☒ | A full description of the statistical parameters including central tendency (e.g. means) or other basic estimates (e.g. regression coefficient) AND variation (e.g. standard deviation) or associated estimates of uncertainty (e.g. confidence intervals) |
| ☐ | ☒ | For null hypothesis testing, the test statistic (e.g. *F*, *t*, *r*) with confidence intervals, effect sizes, degrees of freedom and *P* value noted<br>*Give P values as exact values whenever suitable.* |
| ☒ | ☐ | For Bayesian analysis, information on the choice of priors and Markov chain Monte Carlo settings |
| ☒ | ☐ | For hierarchical and complex designs, identification of the appropriate level for tests and full reporting of outcomes |
| ☒ | ☐ | Estimates of effect sizes (e.g. Cohen's *d*, Pearson's *r*), indicating how they were calculated |

*Our web collection on statistics for biologists contains articles on many of the points above.*

## Software and code

Policy information about availability of computer code

| | |
|---|---|
| Data collection | SpectraMax iD3 Multi-Mode Microplate Reader (Molecular Devices); LSR-II, LSRFortessa, LSR Fortessa X20 and FACSymphony A5 flow cytometers (BD Biosciences); FACSAria and Fusion cell sorters (BD Biosciences); Amnis ImageStreamx MKII; Nikon Ti2/Yokogawa CSU-W1 spinning disk confocal microscope; UVP ChemStudio (Analytik Jena) |
| Data analysis | GraphPad Prism (Version 10); FlowJo (Versions 10.7, 10.8 and 10.9); IMGT/V-Quest (https://www.imgt.org/IMGT_vquest/input); IgBlast (https://www.ncbi.nlm.nih.gov/igblast/); INSPIRE (v200.1.681.0); IDEAS v 6.3 software and standard ImageStream analysis wizards and tools/ algorithms; ImageJ |

For manuscripts utilizing custom algorithms or software that are central to the research but not yet described in published literature, software must be made available to editors and reviewers. We strongly encourage code deposition in a community repository (e.g. GitHub). See the Nature Portfolio guidelines for submitting code & software for further information.

## Data

Policy information about availability of data

All manuscripts must include a data availability statement. This statement should provide the following information, where applicable:

- Accession codes, unique identifiers, or web links for publicly available datasets
- A description of any restrictions on data availability
- For clinical datasets or third party data, please ensure that the statement adheres to our policy

The BCR sequencing data have been deposited in the NLM/NCBI/SRA under accession code PRJNA1054624 (https://www.ncbi.nlm.nih.gov/sra/?term=PRJNA1054624). Properties of antibodies reported in this study are summarized in Supplementary Data 1. Source data are provided with this paper.

## Research involving human participants, their data, or biological material

Policy information about studies with human participants or human data. See also policy information about sex, gender (identity/presentation), and sexual orientation and race, ethnicity and racism.

| | |
|---|---|
| Reporting on sex and gender | *Use the terms sex (biological attribute) and gender (shaped by social and cultural circumstances) carefully in order to avoid confusing both terms. Indicate if findings apply to only one sex or gender; describe whether sex and gender were considered in study design; whether sex and/or gender was determined based on self-reporting or assigned and methods used.*<br>*Provide in the source data disaggregated sex and gender data, where this information has been collected, and if consent has been obtained for sharing of individual-level data; provide overall numbers in this Reporting Summary. Please state if this information has not been collected.*<br>*Report sex- and gender-based analyses where performed, justify reasons for lack of sex- and gender-based analysis.* |
| Reporting on race, ethnicity, or other socially relevant groupings | *Please specify the socially constructed or socially relevant categorization variable(s) used in your manuscript and explain why they were used. Please note that such variables should not be used as proxies for other socially constructed/relevant variables (for example, race or ethnicity should not be used as a proxy for socioeconomic status).*<br>*Provide clear definitions of the relevant terms used, how they were provided (by the participants/respondents, the researchers, or third parties), and the method(s) used to classify people into the different categories (e.g. self-report, census or administrative data, social media data, etc.)*<br>*Please provide details about how you controlled for confounding variables in your analyses.* |
| Population characteristics | *Describe the covariate-relevant population characteristics of the human research participants (e.g. age, genotypic information, past and current diagnosis and treatment categories). If you filled out the behavioural & social sciences study design questions and have nothing to add here, write "See above."* |
| Recruitment | *Describe how participants were recruited. Outline any potential self-selection bias or other biases that may be present and how these are likely to impact results.* |
| Ethics oversight | *Identify the organization(s) that approved the study protocol.* |

Note that full information on the approval of the study protocol must also be provided in the manuscript.

# Field-specific reporting

Please select the one below that is the best fit for your research. If you are not sure, read the appropriate sections before making your selection.

☒ Life sciences ☐ Behavioural & social sciences ☐ Ecological, evolutionary & environmental sciences

For a reference copy of the document with all sections, see nature.com/documents/nr-reporting-summary-flat.pdf

# Life sciences study design

All studies must disclose on these points even when the disclosure is negative.

| | |
|---|---|
| Sample size | The sample sizes were not predetermined using statistical methods but were based on previous experience with similar experiments. We used one to three mice per experimental group for all mouse experiments, and the experiments were repeated two to three times. The sample size and the number of independent experiments are indicated in the figure legends. For testing B cell receptor properties, at least 30 monoclonal antibodies were cloned and produced from each B cell compartment which is also based on prior experience. These sample sizes are sufficient because they revealed significant differences for some group comparisons. |
| Data exclusions | No data were excluded from the analyses unless indicated in methods or figure legends. |
| Replication | All mouse experiments were performed two to three times independently. The sample size and number of independent experiments are indicated in figure legends. Monoclonal antibodies were derived from two to three independent single-cell sorts. Positive reactivity of monoclonal antibodies was confirmed by at least two independent experiments or by two independent methods. The experimental findings were reliably reproduced. |

| | |
|---|---|
| Randomization | Randomization was not relevant to the experiments comparing cell populations within animals of the same genotype. For experiments comparing different genotypes or different treatments, mice were age matched. |
| Blinding | No blinding was performed as mice were grouped by genotype. |

# Reporting for specific materials, systems and methods

We require information from authors about some types of materials, experimental systems and methods used in many studies. Here, indicate whether each material, system or method listed is relevant to your study. If you are not sure if a list item applies to your research, read the appropriate section before selecting a response.

## Materials & experimental systems

| n/a | Involved in the study | |
|---|---|---|
| ☐ | ☒ | Antibodies |
| ☐ | ☒ | Eukaryotic cell lines |
| ☐ | ☐ | Palaeontology and archaeology |
| ☐ | ☒ | Animals and other organisms |
| ☐ | ☐ | Clinical data |
| ☐ | ☐ | Dual use research of concern |
| ☐ | ☐ | Plants |

## Methods

| n/a | Involved in the study | |
|---|---|---|
| ☐ | ☐ | ChIP-seq |
| ☐ | ☒ | Flow cytometry |
| ☐ | ☐ | MRI-based neuroimaging |

## Antibodies

Antibodies used

Monoclonal antibodies:
Active caspase-3 (C92-605 clone; Rabbit IgG), AlexaFluor647, BD #560626; 1:40
Bcl-2 (10C4 clone; Mouse IgG1), eFluor450, ThermoFisher #48-6992-42; 1:20
CD3ε (145-2C11 clone; Armenian Hamster IgG), PE, BD #553064; 1:80
CD4 (RM4-5 clone; Rat IgG2a), Biotin, BioLegend #100508; 1:200
CD4 (RM4-5 clone; Rat IgG2a), APC-eFluor780, ThermoFisher #47-0042-82; 1:200
CD8α (53-6.7 clone; Rat IgG2a), Biotin, BD #553029; 1:200
CD8α (53-6.7 clone; Rat IgG2a), APC-eFluor780, ThermoFisher #47-0081-82; 1:200
CD16/CD32 (2.4G2 clone; Rat IgG2b), Unlabeled, Produced and purified by the EIB Flow Cytometry Core
CD19 (6D5 clone; Rat IgG2a), AlexaFluor594, BioLegend #115552; 1:800
CD19 (6D5 clone; Rat IgG2a), BV605, BioLegend #115540; 1:200
CD21/CD35 (7G6 clone; Rat IgG2b), BUV805, BD #741961; 1:800
CD21/CD35 (7G6 clone; Rat IgG2b), PerCP-Cy5.5, BD #562797; 1:200
CD21/CD35 (7G6 clone; Rat IgG2b), BV510, BD #747764; 1:200
CD23 (B3B4 clone; Rat IgG2a), BV786, BD #563988; 1:200
CD38 (90 clone; Rat IgG2a), AlexaFluor700, ThermoFisher #56-0381-82; 1:200
CD43 (S7 clone; Rat IgG2a), PE-Cy7, BD #562866; 1:160
CD43 (S7 clone; Rat IgG2a), PerCP-Cy5.5, BD #562865; 1:25
CD45R/B220 (RA3-6B2 clone; Rat IgG2a), AlexaFluor594, BioLegend #103254; 1:400
CD45R/B220 (RA3-6B2 clone; Rat IgG2a), PE-Cy7, ThermoFisher #25-0452-82; 2µg i.v.
CD45R/B220 (RA3-6B2 clone; Rat IgG2a), AlexaFluor647, ThermoFisher #RM2621; 1:400
CD93/AA4.1 (AA4.1 clone; Rat IgG2b), APC, ThermoFisher #17-5892-83; 1:100
CD93/AA4.1 (AA4.1 clone; Rat IgG2b), PE, BioLegend #136504, 1:40
CD95/Fas (Jo2 clone; Armenian Hamster IgG), R718, BD #752226; 1:3200
CD95/Fas (Jo2 clone; Armenian Hamster IgG), PE-Cy7, BD #557653; 1:800
CD138 (281-2 clone; Rat IgG2a), BV711, BioLegend #142519; 1:8000
CD267/TACI (ebio8F10-3; Rat IgG2a), PE, ThermoFisher #12-5942-81; 1:200
F4/80 (BM8 clone; Rat IgG2a), Biotin, ThermoFisher #13-4801-85; 1:100
F4/80 (BM8 clone; Rat IgG2a), APC-eFluor780, ThermoFisher #47-4801-82; 1:100
GL7 (GL7 clone; Rat IgM), Biotin, BioLegend #144616; 1:3200
GL7 (GL7 clone; Rat IgM), eFluor660, BioLegend #144616; 1:400
IgD (11-26c.2a clone; Rat IgG2a), BUV395, BD #564274; 1:100
IgM (RMM-1 clone; Rat IgG2a), BV421, BioLegend #406517; 1:10
IgM (II/41 clone; Rat IgG2a), PE-Cy7, ThermoFisher #25-5790-82; 1:100
IgM (II/41 clone; Rat IgG2a), eFluor660, ThermoFisher #50-5790-82; 1:25
Ly-6G (1A8 clone; Rat IgG2a), Biotin, BioLegend #127604; 1:100
NK1.1 (PK136 clone; Mouse IgG2a), Biotin, BD #553163; 1:200
NK1.1 (PK136 clone; Mouse IgG2a), APC-eFluor780, ThermoFisher #47-5941-82; 1:200
TER-119 (TER-119 clone; Rat IgG2b), Biotin, ThermoFisher #13-5921-82; 1:400

Polyclonal antibodies:
Alexa Fluor® 647 AffiniPure F(ab')$_2$ Fragment Goat Anti-Human IgG, Fcγ fragment specific (min X Bov, Hrs, Ms Sr Prot), JacksonImmunoResearch #109-606-098, 1µg/ml
Peroxidase AffiniPure Goat Anti-Human IgG, Fcγ fragment specific (min X Bov, Hrs, Ms Sr Prot), JacksonImmunoResearch

| | |
|---|---|
| | #109-035-098, 1:1000 (Polyreactivity) or 1:5000 (Dot blot assay) |
| Validation | All antibodies are commonly used clones, commercially available and validated both by the manufacturer, through citations in the scientific literature, or through use in previous experiments. Validation materials for each antibody can be accessed on the respective manufacturer's homepage. We additionally validated all antibodies prior to use in our study by performing titration on primary mouse bone marrow or spleen cells as appropriate, starting with the recommended dilution. |

## Eukaryotic cell lines

Policy information about cell lines and Sex and Gender in Research

| | |
|---|---|
| Cell line source(s) | Freestyle 293-F cells (ThermoFisher, R79007) |
| Authentication | The cell line was directly purchased from ThermoFisher and not authenticated |
| Mycoplasma contamination | The cell line was directly purchased from ThermoFisher and not tested for Mycoplasma contamination |
| Commonly misidentified lines (See ICLAC register) | No commonly misidentified cell line was used in this study |

## Palaeontology and Archaeology

| | |
|---|---|
| Specimen provenance | *Provide provenance information for specimens and describe permits that were obtained for the work (including the name of the issuing authority, the date of issue, and any identifying information). Permits should encompass collection and, where applicable, export.* |
| Specimen deposition | *Indicate where the specimens have been deposited to permit free access by other researchers.* |
| Dating methods | *If new dates are provided, describe how they were obtained (e.g. collection, storage, sample pretreatment and measurement), where they were obtained (i.e. lab name), the calibration program and the protocol for quality assurance OR state that no new dates are provided.* |

☐ Tick this box to confirm that the raw and calibrated dates are available in the paper or in Supplementary Information.

| | |
|---|---|
| Ethics oversight | *Identify the organization(s) that approved or provided guidance on the study protocol, OR state that no ethical approval or guidance was required and explain why not.* |

Note that full information on the approval of the study protocol must also be provided in the manuscript.

## Animals and other research organisms

Policy information about studies involving animals; ARRIVE guidelines recommended for reporting animal research, and Sex and Gender in Research

| | |
|---|---|
| Laboratory animals | Rosa26-INDIA mice (PMID: 28935768) and Eμ-Bcl2-tg mice (PMID: 1924327) were both on a C57Bl/6J background and a kind gift by Dr. Michel Nussenzweig (The Rockefeller University). Nur77-GFP mice (PMID: 22902503) were on a C57Bl/6J background and a kind gift by Dr. Richard Hodes (NCI). C57Bl/6J mice were purchased from Jackson Laboratories (Strain #664). Rosa26-INDIA mice in Figures 1 and 3 were 8.3 to 13.9 weeks old. Rosa26-INDIA mice in Figure 2 were 6.9 to 12.6 weeks old. Rosa26-INDIA mice used for single-cell sorting in Figure 4 were 7.4 to 13.4 weeks old. Nur77-GFP mice in Figure 5a-d were 9.7 to 17.0 weeks old, and Nur77-GFP x Eμ-Bcl2-tg mice in the same Figure were 12.7 to 17.0 weeks old. Nur77-GFP mice in Figure 5e-j were 12.3 to 26.9 weeks old. |
| Wild animals | This study did not involve wild animals |
| Reporting on sex | The experiments in this study contain mice from both sexes and therefore findings do not apply to only one sex. Figures 1 and 3 used five male (bone marrow, blood) or four male (spleen) Rosa26-INDIA mice and one female Rosa26-INDIA mouse. Figure 2 used four male Rosa26-INDIA mice and one female Rosa26-INDIA mouse. Single-cell sorting in Figure 4 used two male and four female Rosa26-INDIA mice. Figure 5a-d used zero male and seven female Nur77-GFP mice, four male and three female Nur77-GFP x Eμ-Bcl2-tg mice. Figure 5e-j used three male and two female Nur77-GFP mice. |
| Field-collected samples | This study did not involve samples collected from the field |
| Ethics oversight | All animal procedures reported in this study that were performed by NCI-CCR affiliated staff were approved by the NCI Animal Care and Use Committee and in accordance with federal regulatory requirements and standards. All components of the intramural NIH ACU program are accredited by AAALAC International. |

Note that full information on the approval of the study protocol must also be provided in the manuscript.

# Clinical data

Policy information about clinical studies

All manuscripts should comply with the ICMJE guidelines for publication of clinical research and a completed CONSORT checklist must be included with all submissions.

| | |
|---|---|
| Clinical trial registration | *Provide the trial registration number from ClinicalTrials.gov or an equivalent agency.* |
| Study protocol | *Note where the full trial protocol can be accessed OR if not available, explain why.* |
| Data collection | *Describe the settings and locales of data collection, noting the time periods of recruitment and data collection.* |
| Outcomes | *Describe how you pre-defined primary and secondary outcome measures and how you assessed these measures.* |

# Dual use research of concern

Policy information about dual use research of concern

## Hazards

Could the accidental, deliberate or reckless misuse of agents or technologies generated in the work, or the application of information presented in the manuscript, pose a threat to:

| No | Yes | |
|---|---|---|
| ☒ | ☐ | Public health |
| ☒ | ☐ | National security |
| ☒ | ☐ | Crops and/or livestock |
| ☒ | ☐ | Ecosystems |
| ☒ | ☐ | Any other significant area |

## Experiments of concern

Does the work involve any of these experiments of concern:

| No | Yes | |
|---|---|---|
| ☒ | ☐ | Demonstrate how to render a vaccine ineffective |
| ☒ | ☐ | Confer resistance to therapeutically useful antibiotics or antiviral agents |
| ☒ | ☐ | Enhance the virulence of a pathogen or render a nonpathogen virulent |
| ☒ | ☐ | Increase transmissibility of a pathogen |
| ☒ | ☐ | Alter the host range of a pathogen |
| ☒ | ☐ | Enable evasion of diagnostic/detection modalities |
| ☒ | ☐ | Enable the weaponization of a biological agent or toxin |
| ☒ | ☐ | Any other potentially harmful combination of experiments and agents |

# Plants

| | |
|---|---|
| Seed stocks | *Report on the source of all seed stocks or other plant material used. If applicable, state the seed stock centre and catalogue number. If plant specimens were collected from the field, describe the collection location, date and sampling procedures.* |
| Novel plant genotypes | *Describe the methods by which all novel plant genotypes were produced. This includes those generated by transgenic approaches, gene editing, chemical/radiation-based mutagenesis and hybridization. For transgenic lines, describe the transformation method, the number of independent lines analyzed and the generation upon which experiments were performed. For gene-edited lines, describe the editor used, the endogenous sequence targeted for editing, the targeting guide RNA sequence (if applicable) and how the editor was applied.* |
| Authentication | *Describe any authentication procedures for each seed stock used or novel genotype generated. Describe any experiments used to assess the effect of a mutation and, where applicable, how potential secondary effects (e.g. second site T-DNA insertions, mosiacism, off-target gene editing) were examined.* |

# ChIP-seq

## Data deposition

☐ Confirm that both raw and final processed data have been deposited in a public database such as GEO.

☐ Confirm that you have deposited or provided access to graph files (e.g. BED files) for the called peaks.

| | |
|---|---|
| Data access links<br>*May remain private before publication.* | *For "Initial submission" or "Revised version" documents, provide reviewer access links. For your "Final submission" document, provide a link to the deposited data.* |
| Files in database submission | *Provide a list of all files available in the database submission.* |
| Genome browser session<br>(e.g. UCSC) | *Provide a link to an anonymized genome browser session for "Initial submission" and "Revised version" documents only, to enable peer review. Write "no longer applicable" for "Final submission" documents.* |

## Methodology

| | |
|---|---|
| Replicates | *Describe the experimental replicates, specifying number, type and replicate agreement.* |
| Sequencing depth | *Describe the sequencing depth for each experiment, providing the total number of reads, uniquely mapped reads, length of reads and whether they were paired- or single-end.* |
| Antibodies | *Describe the antibodies used for the ChIP-seq experiments; as applicable, provide supplier name, catalog number, clone name, and lot number.* |
| Peak calling parameters | *Specify the command line program and parameters used for read mapping and peak calling, including the ChIP, control and index files used.* |
| Data quality | *Describe the methods used to ensure data quality in full detail, including how many peaks are at FDR 5% and above 5-fold enrichment.* |
| Software | *Describe the software used to collect and analyze the ChIP-seq data. For custom code that has been deposited into a community repository, provide accession details.* |

# Flow Cytometry

## Plots

Confirm that:

☒ The axis labels state the marker and fluorochrome used (e.g. CD4-FITC).

☒ The axis scales are clearly visible. Include numbers along axes only for bottom left plot of group (a 'group' is an analysis of identical markers).

☐ All plots are contour plots with outliers or pseudocolor plots.

☒ A numerical value for number of cells or percentage (with statistics) is provided.

## Methodology

Sample preparation

For bone marrow sinusoid B cell labeling, mice were injected intravenously with 2µg PE-Cy7-conjugated anti-CD45R/B220 antibody (Thermo Fisher Scientific, 25-0452-82) 2 min prior to euthanasia. For Rosa26-INDIA experiments, euthanized mice, buffers, and consumables contacting cells were pre-chilled and maintained on ice to minimize de novo apoptosis during cell isolation, staining and acquisition. All centrifugations and cell sorting were done at 4°C.
Immediately after euthanasia peripheral blood was drawn from the inferior vena cava, mixed with 10mM EDTA to prevent coagulation and added to 10ml ACK lysis buffer (Quality Biological, 118-156-101). After incubation for 5 min on ice, cells were centrifuged for 7 min at 1300 RPM and washed with 10ml of FACS buffer. Bone marrow was flushed out from femurs and tibiae. Single-cell suspensions were created from bone marrow and spleens by forcing the tissue through 70 µm cell strainers (Corning, 352350). For spleen, erythrocytes were lysed by resuspending pellets in 1ml ACK lysis buffer (Quality Biological, 118-156-101) and incubating for 1 min on ice, followed by washing with FACS buffer. Blood leukocytes, bone marrow and spleen cell suspensions were then transferred to 96-well round bottom plates. All centrifugations were performed for 3 min at 1300 RPM.
For live cell staining, Fc receptors were first blocked for 15 min on ice with rat anti-mouse CD16/CD32 antibody (2.4G2, produced and purified by the EIB Flow Cytometry Core). After centrifugation, cells were stained with antibodies to surface antigens for 45 min at 4°C and washed three times with FACS buffer. If applicable, cells were then stained with fluorescent streptavidin for 10 min at 4°C and washed three times. For some experiments, cells were first stained with Far-Red fluorescent FLICA 660 VAD diluted in 1xPBS for 1h at 4°C or 37°C prior to the staining steps above. For some experiments, cells were stained with AlexaFluor647-conjugated Annexin-V (Invitrogen, A23204) in 1x Annexin binding buffer (ThermoFisher, V13246) as the final step. Cells were resuspended in FACS buffer containing 0.2µg/ml propidium iodide (PI) (Sigma-Aldrich, P4170) or 0.1µg/ml DAPI (Sigma-Aldrich, D9542), or in 1x Annexin binding buffer contain DAPI, prior to acquisition to exclude dead/necrotic cells.
For intracellular staining and for some live cell experiments where PI and DAPI were not compatible with the staining panel, cells were washed twice with PBS and stained using the Zombie NIR or Zombie UV Fixable Viability Kit (Biolegend, 423106 or

423108, diluted 1:500 in PBS) for 30 min at 4°C prior to Fc blocking and staining of surface antigens. For intracellular staining, cells were then washed with PBS and incubated with Fixation/Permeabilization solution (BD Biosciences, 51-2090KZ) for 30 min at 4°C, except for simultaneous analysis of aCasp3+ and FRETneg cells where fixation for 20min at room temperature was found to better preserve INDIA fluorescence. Cells were washed two times with Perm/Wash buffer (BD Biosciences, 51-2091KZ) and then incubated with antibodies against intracellular antigens diluted in Perm/Wash buffer for 45 min at 4°C. After three washes with Perm/Wash buffer, cells were resuspended in FACS buffer.

Instrument

LSR-II, LSRFortessa, LSR Fortessa X20 and FACSymphony A5 flow cytometers (BD Biosciences); FACSAria and Fusion cell sorters (BD Biosciences)

Software

FlowJo (Versions 10.7, 10.8 and 10.9)

Cell population abundance

Post-sort purity of FRET- and FRET+ B cells was determined by subsequent intracellular active caspase-3 staining followed by flow cytometry and is depicted in Supplementary Fig. 1b. Post-sort purity of B cells was additionally determined by recording events from pre-sort and post-sort samples on the FACS sorter, and was >99%.

Gating strategy

Also see Supplementary Fig. 1c-e and Supplementary Fig. 5. Debris were excluding using FSC-A/SSC-A gates and aggregates were excluded using FSC-W/SSC-A gates. Dead cells were excluded by gating on cells lacking staining with DAPI, PI, Zombie-NIR or Zombie-UV.

In Fig. 1, bone marrow B cells were gated CD45R/B220+mRuby2+DAPI(neg) and CD43+IgD(neg)IgM(neg) (Fr. B/C), CD43(neg)IgD(neg)IgM(neg) (Fr. D), CD43(neg)IgD(neg/lo)IgM+ (Fr. E) or CD43(neg)IgD(hi)IgM+ (Fr. F). Blood and spleen B cells were gated CD45R/B220+mRuby2+DAPI(neg)Lineage(CD4, CD8α, F4/80, NK1.1, Ly-6G, Ter-119)negCD95(neg) and IgD(lo)IgM(hi)CD21(neg/lo) (transitional, T), IgD(lo)IgM(hi)CD21(neg)CD23(neg) (transitional 1, T1), IgD(lo)IgM(hi)CD21(lo)CD23+ (transitional 2, T2), IgD(lo)IgM(hi)CD21(hi) (MZ), IgD+IgM+CD23+CD93/AA4.1(neg) (follicular, FO), or IgD+IgM+CD23+CD93/AA4.1+IgM(low) (anergic, T3). The full gating strategy is shown in Supplementary Fig. 1c-e. We recommend avoiding CD93/AA4.1 if possible because this marker is relatively weakly expressed on transitional B cells and tends to be further downregulated during apoptosis resulting in incomplete capturing of all FRET(neg) cells.
Gating in Fig. 2 was performed as in Fig. 1 except intravascular anti-CD45R/B220-PE-Cy7 was used to label bone marrow sinusoids, CD19 was used to stain B cells instead of CD45R/B220, dead cells were excluded using Zombie NIR, and FO B cells were gated IgD(hi)IgM+.
Gating in Supplementary Fig. 1j,k and Supplementary Fig. 2 was as in Fig.1 but without CD93/AA4.1 and distinguishing T3 cells.
Gating in Supplementary Fig. 4 was CD45R/B220+mRuby2+DAPI(neg)Lineage(CD4, CD8α, F4/80, NK1.1, Ly-6G, Ter-119)negCD95(neg) and CD93/AA4.1+CD23(neg) (transitional 1 B cells, T1) or CD93/AA4.1(neg)CD23+ (mature follicular B cells, FO). IgM(lo) and IgM(hi) subsets were distinguished.
For Rosa26INDIA bulk sorting in Supplementary Fig. 1b, the following gating was done on bone marrow and spleen cells: B220+Lineage(CD4, CD8α, NK1.1, F4/80, Ly-6G, Ter-119)negDAPI(neg)mRuby2+ and FRET+ or FRET(neg). In case of splenic B cells, GL7(hi) GCB cells were additionally excluded.

Gating for other experiments was performed as follows:
In Fig. 5, spleen B cells were gated PI(neg)CD138(lo)TACI(lo)CD19+Fas(neg) (Fig. 5a-d) or Zombie-NIR(neg)Lin(CD4,CD8α,NK1.1,Ly-6G,Ter-119,F4/80)negCD19+Fas(neg)(Fig. 5e-j) and AA4.1+CD21(neg)CD23(neg) (transitional 1 B cells, T1), CD93/AA4.1+CD21(lo)CD23+ (transitional 2/3 B cells, T2/3) or CD93/AA4.1(neg)GL7(neg)CD21+CD23+ (mature follicular B cells, FO).

☒ Tick this box to confirm that a figure exemplifying the gating strategy is provided in the Supplementary Information.

# Magnetic resonance imaging

## Experimental design

Design type

*Indicate task or resting state; event-related or block design.*

Design specifications

*Specify the number of blocks, trials or experimental units per session and/or subject, and specify the length of each trial or block (if trials are blocked) and interval between trials.*

Behavioral performance measures

*State number and/or type of variables recorded (e.g. correct button press, response time) and what statistics were used to establish that the subjects were performing the task as expected (e.g. mean, range, and/or standard deviation across subjects).*

## Acquisition

| | |
|---|---|
| Imaging type(s) | *Specify: functional, structural, diffusion, perfusion.* |
| Field strength | *Specify in Tesla* |
| Sequence & imaging parameters | *Specify the pulse sequence type (gradient echo, spin echo, etc.), imaging type (EPI, spiral, etc.), field of view, matrix size, slice thickness, orientation and TE/TR/flip angle.* |
| Area of acquisition | *State whether a whole brain scan was used OR define the area of acquisition, describing how the region was determined.* |

Diffusion MRI ☐ Used ☐ Not used

## Preprocessing

| | |
|---|---|
| Preprocessing software | *Provide detail on software version and revision number and on specific parameters (model/functions, brain extraction, segmentation, smoothing kernel size, etc.).* |
| Normalization | *If data were normalized/standardized, describe the approach(es): specify linear or non-linear and define image types used for transformation OR indicate that data were not normalized and explain rationale for lack of normalization.* |
| Normalization template | *Describe the template used for normalization/transformation, specifying subject space or group standardized space (e.g. original Talairach, MNI305, ICBM152) OR indicate that the data were not normalized.* |
| Noise and artifact removal | *Describe your procedure(s) for artifact and structured noise removal, specifying motion parameters, tissue signals and physiological signals (heart rate, respiration).* |
| Volume censoring | *Define your software and/or method and criteria for volume censoring, and state the extent of such censoring.* |

## Statistical modeling & inference

| | |
|---|---|
| Model type and settings | *Specify type (mass univariate, multivariate, RSA, predictive, etc.) and describe essential details of the model at the first and second levels (e.g. fixed, random or mixed effects; drift or auto-correlation).* |
| Effect(s) tested | *Define precise effect in terms of the task or stimulus conditions instead of psychological concepts and indicate whether ANOVA or factorial designs were used.* |

Specify type of analysis: ☐ Whole brain ☐ ROI-based ☐ Both

| | |
|---|---|
| Statistic type for inference<br>(See Eklund et al. 2016) | *Specify voxel-wise or cluster-wise and report all relevant parameters for cluster-wise methods.* |
| Correction | *Describe the type of correction and how it is obtained for multiple comparisons (e.g. FWE, FDR, permutation or Monte Carlo).* |

## Models & analysis

| n/a | Involved in the study |
|---|---|
| ☐ | ☐ Functional and/or effective connectivity |
| ☐ | ☐ Graph analysis |
| ☐ | ☐ Multivariate modeling or predictive analysis |

| | |
|---|---|
| Functional and/or effective connectivity | *Report the measures of dependence used and the model details (e.g. Pearson correlation, partial correlation, mutual information).* |
| Graph analysis | *Report the dependent variable and connectivity measure, specifying weighted graph or binarized graph, subject- or group-level, and the global and/or node summaries used (e.g. clustering coefficient, efficiency, etc.).* |
| Multivariate modeling and predictive analysis | *Specify independent variables, features extraction and dimension reduction, model, training and evaluation metrics.* |
